# Epitope characterization of the protective monoclonal antibody VN04-2 shows broadly neutralizing activity against highly pathogenic H5N1

**DOI:** 10.1186/1743-422X-5-80

**Published:** 2008-07-11

**Authors:** Angeline PC Lim, Steven KK Wong, Annie HY Chan, Conrad EZ Chan, Eng Eong Ooi, Brendon J Hanson

**Affiliations:** 1 Defence Medical and Environmental Research Institute, DSO National Laboratories, 27 Medical Dr., 117510, Singapore

## Abstract

The monoclonal antibody VN04-2 was previously shown to protect mice against lethal A/Vietnam/1203/04 H5N1 virus challenge when administered pre- and post-infection. In this study, we characterized the binding requirements of this antibody using direct binding to hemagglutinin and neutralization assays with H5N1 virus-like particles (H5N1-VLP) of eight recent H5N1 strains representing the major mutations within the 140s antigenic loop. Binding was clade independent and 3 mutations within this antigenic region are required before escape is possible, suggesting that apart from the H5N1 viruses circulating in Indonesia, VN04-2 may provide protection against H5N1 viruses from all other regions.

## Findings

In 1997, human disease was first reported due to direct transmission from poultry of highly pathogenic avian influenza A virus (HPAI) of the subtype H5N1, resulting in the death of 6 of the 18 infected individuals [1-3]. Increased geographical distribution (H5N1 has been reported in a variety of birds from over 50 countries) coupled with continued evolution of H5N1 viruses and an immunologically naïve human population highlight the pandemic potential of these viruses [4,5]. Virus spread among the human population has been limited and largely remains the result of direct bird-to-human transmission. As of mid-January 2008, there have been 349 reported cases of human H5N1 infection with a high mortality rate resulting in the death of 216 individuals [6]. Recently, we and others have reported therapeutic efficacy of passive immunization in a HPAI H5N1 mouse model with either humanized mouse mAb, equine F(ab')_2_, or human mAb, highlighting its potential as a viable treatment option in human cases of H5N1 [7-9]. Indeed, survival of a person infected with HPAI H5N1 has been reported after treatment with convalescent plasma [10]. A potential drawback to the use of specific mAb is that the high mutation rate of influenza viruses particularly in the antigenic regions means that escape from the protective effect of these antibodies may be rapid. In the case of our humanized mAb VN04-2 (also termed 15A3) specific for the 140s antigenic loop, hemagglutination inhibition (HI) assay data suggested an absolute requirement for lysine at position 140 [8,11]. However, mutation of H5N1 viruses outside of antibody binding sites have been shown to negatively affect the performance of the viruses in HI assays, suggesting that in some cases a negative HI assay result may be more a limitation of the assay rather than lack of antibody binding [12]. Here we evaluated binding of VN04-2 to a variety of H5 hemagglutinins (HA) independent of the HI assay, to determine the actual effects mutations in this region of the HA gene has on antibody binding and the utility of the antibody for protection against recently circulating H5N1 viruses.

The mAb VN04-2 was raised against the HA of A/Vietnam/1203/04, therefore to select the HAs to be used in this study, we aligned all the HA sequences from H5N1 viruses isolated throughout 2005 and 2006, that were deposited into the Influenza Virus Resource and was maintained by NCBI, against this HA [13]. Focusing on mutation within the 140s loop antigenic region, the HA sequences could be divided into eight groups, and a representative of each of these was selected to be used in the antibody binding analysis (Table 1). The cDNAs encoding the HA1 subunits of the selected HAs were produced by a combination of PCR based methods and the fidelity of each clone was confirmed by sequencing. In order to produce the HA proteins, we used the recombinant baculovirus expression method described for determination of the H5 HA structure, where the transmembrane domain had been replaced by the 'foldon' trimerization sequence, allowing for expression of soluble HA trimers which could be purified by virtue of the carboxyl terminal hexa-histidine tag [14]. Following introduction of the foldon sequence into the HA2 of A/Vietnam/1203/04 and insertion into plasmids containing each of the HA1s listed in table 1, recombinant baculoviruses were produced and used to infect Sf9 insect cells. All nine of the HA-foldons could be purified from culture medium using talon affinity resin and cleavage into HA1 and HA2 subunits with trypsin indicated that the proteins were correctly folded (data not shown).

**Table 1:** Position of mutation in the selected HA1s compared to A/Vietnam/1203/04^*a*^

		Amino acid position											
		
Virus	Clade^*b*^				140s Loop					150s Loop			
							
		94	124	129	136	137	138	140	141	154	155	156	189
A/Vietnam/1203/04	1	D	S	L	P	Y	Q	K	S	N	S	T	K
A/DK/Vietnam/376/05	1	**.**	**.**	**.**	**.**	**.**	**.**	N	P	**.**	**.**	**.**	**.**
A/BhGs/Qing Hai/65/05	2.2	N	D	S	**.**	**.**	**.**	R	**.**	**.**	N	A	R
A/CK/Ivory Coast/1787/06	2.2	N	D	S	**.**	H	**.**	R	**.**	D	N	A	R
A/Zhe Jiang/16/06	2.3.4	N	D	S	**.**	**.**	**.**	T	P	**.**	N	**.**	**.**
A/DK/Guangzhou/20/05	9	**.**	N	S	**.**	**.**	L	**.**	P	**.**	N	A	**.**
A/Indonesia/CDC597/06	2.1.2	N	D	**.**	**.**	**.**	L	R	**.**	**.**	N	**.**	R
A/Indonesia/5/05	2.1.3	S	D	S	**.**	**.**	L	S	P	**.**	**.**	**.**	R
A/CK/Indonesia/R60/05	2.1.1	N	D	S	S	**.**	L	D	P	**.**	**.**	A	R

^*a *^residues similar to A/Vietnam/1203/04 are marked by a period

^*b *^Clade nomenclature as suggested by WHO [19]

**Figure 1:**
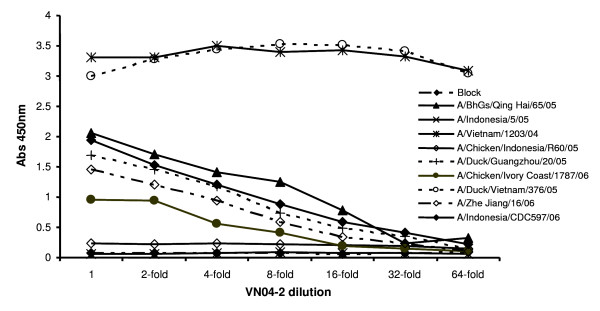
**Affinity of VN04-2 against various HA-foldons determined by ELISA**. Purified HA-foldons from the indicated H5N1 viruses (outlined in table 1) were used to coat ELISA plates and incubated with VN04-2 (1 ug/mL) or its 2 fold serial dilutions, bound antibody was detected with anti-human IgG conjugated to HRP and visualized using TMB. Data shown are the averages from two independent experiments.

To examine the ability of the humanized antibody VN04-2 to bind to the selected HAs, ELISA was performed. Figure 1 shows the level of binding detected with 1 ug/mL VN04-2 antibody and several serial dilutions, after the various HA-foldons were coated onto ELISA plates at 500 ng/well. Highest signal was observed with the immunogen HA from A/Vietnam/1203/04, while the HAs from A/Indonesia/5/05 and A/Ck/Indonesia/R60/05 were unable to bind VN04-2 at all, suggesting that 3 mutations within the 140s loop antigenic site are required to escape antibody binding, a conclusion supported as the remaining HAs showed binding of VN04-2 albeit at varying degrees. Interestingly, the HA from A/Dk/Vietnam/376/05 which only contains mutations within the 140s loop showed similar binding characteristics to that of A/Vietnam/1203/04. Therefore, the amino acids within the 140s loop may be the main determinants of antibody binding for VN04-2, but residues outside of this region may also contribute to the overall antibody binding affinity. Previous studies with H3N2 influenza A virus have indicated that antibodies against the 140s loop antigenic site with association constants (K_*A*_) in the 10^6 ^M^-1 ^range can bind HA by ELISA and exhibit neutralizing efficacy [15]. Therefore to definitively measure the actual binding kinetics of VN04-2 to the various HAs that showed binding in the ELISA assay, we also measured the affinity of VN04-2 for the various HA-foldons using Biacore SPR analysis. The antibody showed a range of association constants for the HAs with the highest calculated against A/Vietnam/1203/04 (2.63 × 10^8 ^M^-1^) and the lowest calculated against the lowest ELISA binding A/CK/Ivory Coast/1787/06 (1.93 × 10^7 ^M^-1^), indicating good agreement between the ELISA data and the actual antibody/HA K_*A *_(Table 2) Together, these results deduce that the absolute requirement for lysine at residue 140, as indicated previously by the HI assay, was most likely due to assay limitations rather than the actual binding properties of the antibody. However, as limited mutation in the 140s antigenic loop and elsewhere lowers the affinity of VN04-2 interaction with HA, we wanted to determine if the lower affinity correlated to a loss of neutralization.

**Table 2:** Equilibrium association (K_*A*_) and dissociation (K_*D*_)constants of VN04-2 with HA

Virus of HA-Foldon	*K*_*A *_(M^-1^)	*K*_*D *_(M)
A/Vietnam/1203/04	2.63 × 10^8^	3.8 × 10^-9^
A/DK/Vietnam/376/05	9.72 × 10^7^	1.03 × 10^-8^
A/BhGs/Qing Hai/65/05	3.08 × 10^7^	3.25 × 10^-8^
A/CK/Ivory Coast/1787/06	1.93 × 10^7^	5.17 × 10^-8^
A/Zhe Jiang/16/06	2.36 × 10^7^	4.24 × 10^-8^
A/DK/Guangzhou/20/05	5.46 × 10^7^	1.83 × 10^-8^
A/Indonesia/CDC597/06	3.38 × 10^7^	2.96 × 10^-8^

Recently, virus-like particles (VLP) built on a retroviral core particle, harboring the surface proteins of Venezuelan equine encephalitis virus and H5N1 have shown their potential as vaccine candidates and also through inclusion of either luciferase or GFP reporter genes, utility as a substitute for live virus in cell based neutralization assays [16-18]. The VLP utilizes the core particle of the moloney murine leukemia virus and as it is non-replicative, is ideally suited for pseudotyping of high containment viruses such as H5N1. To enable expression of H5N1-VLPs, we cloned the HA1 cDNAs described in table 1 together with HA2 of A/Vietnam/1203/04 into the CMV promoter driven expression vector, pXJ and the N1 neuraminidase (NA) of A/Vietnam/1203/04 into pCI (Promega). The plasmids encoding the core particle and GFP reporter gene, pVPack-GP and pFB-hrGFP respectively were purchased from Stratagene. Following introduction of the plasmids into HEK293, the production of H5N1-VLPs was confirmed by immunoblots and observation of GFP in MDCK cells after incubation with the HEK293 culture medium (data not shown).

To determine the ability of VN04-2 to neutralize transduction of the various H5N1-VLPs, HEK293 culture supernatants were incubated with 2 ug/mL VN04-2 for 60 min prior to the addition to MDCK, and 3 days later the number of cells expressing GFP was determined by flow cytometry. As highlighted in table 3, except for the H5N1-VLPs produced using the HAs from A/Indonesia/5/05 and A/Ck/Indonesia/R60/05, VN04-2 was able to neutralize the transduction of all the H5N1-VLPs tested. It is worth noting that HAs which exhibited the lowest affinity for VN04-2 also exhibited less neutralization, indicating a correlation between direct binding affinity and effectiveness of viral neutralization for a known neutralizing antibody. In addition, when we used the culture supernatants incubated with VN04-2 in a HI assay, inhibition was only observed when the H5N1-VLPs HA had aspartic acid residue 94 (Table 3), which is in agreement with the HI data reported by Chen et al, presented in table 1[11]. Taken together the results support the hypothesis that the absolute requirement of lysine at residue 140 was due to a limitation of the assay and not the antibody. While in vitro data does not always reliably predict in vivo efficacy [7]. The demonstrated in vivo efficacy of VN04-2, coupled with the relative insensitivity of this antigenic region to the low pH induced conformational changes of HA, prior to fusion as seen in H3N2 [15]: we believe that in this case, in vitro binding data could be indicative of in vivo efficacy. However, this can only be confirmed with empirical data.

**Table 3:** Determination of VN04-2 neutralization of H5N1-VLPs

	H5N1-VLP^*a*^		Virus^*b*^
		
Virus	Neutralization (%)	HI assay	HI titer
A/Vietnam/1203/04	100	+	12,800
A/DK/Vietnam/376/05	100	+	ND
A/BhGs/Qing Hai/65/05	94	-	<
A/CK/Ivory Coast/1787/06	88	-	200^1^
A/Zhe Jiang/16/06	78	-	ND
A/DK/Guangzhou/20/05	99	+	6,400^2^
A/Indonesia/CDC597/06	99	-	ND
A/Indonesia/5/05	5	-	<^3^
A/CK/Indonesia/R60/05	0	-	ND

^*a *^assays performed using humanized VN04-2 antibody at 2 ug/mL

^*b *^assay data taken from Chen et al [11] performed using the mouse VN04-2 antibody (15A3) of unknown concentration; data obtained with virus exhibiting identical mutations relative to A/Vietnam/1203/04, ^1^MDk/JX/2136/05, ^2^MDk/JX/1653/, and ^3^Ck/Wajo/BBVM/05

In conclusion, our results show that the protective humanized antibody VN04-2 we have previously described is capable of tolerating 3 mutations within its epitope, the 140s loop and that residues outside of this loop while not being major determinants of antibody binding do affect the affinity of the antibody binding to HA. In addition, our results indicate that the previous requirements for VN04-2 binding derived from HI assay data may have been due to assay limitations rather than the actual antibody binding and adds to an increasing amount of evidence questioning the usefulness of HI assays as a measure of neutralization, or for epitope mapping. The HA clones described here were representative of the antigenic drift observed during 2005 and 2006 within this antigenic region, and is still the case for H5N1 strains isolated throughout 2007, suggesting that apart from the H5N1 viruses circulating in Indonesia, VN04-2 may provide protection against H5N1 viruses from all other regions.

## Competing interests

The authors declare that they have no competing interests.

## Authors' contributions

APCL, OEE and BJH conceived the study. APCL and BJH planned the experimental design, performed the baculovirus and VLP work and drafted the manuscript. SKKW participated in the design and performed of HA1 cloning strategies. AHYC and CEZC helped with HA1 cloning and provided general technical assitance. All authors critically reviewed and approved the final manuscript.
